# Problematic consumption of online pornography during the COVID-19 pandemic: clinical recommendations

**DOI:** 10.47626/2237-6089-2020-0090

**Published:** 2021-10-22

**Authors:** Nino Cesar Marchi, Letícia Fara, Luana Gross, Felipe Ornell, Alessandra Diehl, Felix Henrique Paim Kessler

**Affiliations:** 1 Centro de Pesquisa em Álcool e Drogas Hospital de Clínicas de Porto Alegre Universidade Federal do Rio Grande do Sul Porto Alegre RS Brazil Centro de Pesquisa em Álcool e Drogas, Hospital de Clínicas de Porto Alegre (HCPA), Universidade Federal do Rio Grande do Sul (UFRGS), Porto Alegre, RS, Brazil.; 2 Programa de Pós-Graduação em Psiquiatria e Ciências do Comportamento UFRGS Porto Alegre RS Brazil Programa de Pós-Graduação em Psiquiatria e Ciências do Comportamento, UFRGS, Porto Alegre, RS, Brazil.; 3 Universidade Federal de São Paulo São Paulo SP Brazil Universidade Federal de São Paulo (UNIFESP), São Paulo, SP, Brazil.

**Keywords:** Online pornography, pandemics, COVID-19, mental health, clinical features

## Abstract

The coronavirus disease (COVID-19) pandemic is one of the greatest contemporary challenges. Feelings of fear and uncertainty triggered by this pandemic have had noxious effects on people’s mental health. This seems to have increased during quarantine and there is evidence of an intensification of reward-directed behavior. Nevertheless, there are few studies dealing with pornography consumption during this period. The aim of this manuscript is to contextualize this phenomenon during the pandemic and suggest some clinical recommendations on the matter.

## Background

The novel form of coronavirus disease (COVID-19) was initially detected in Wuhan, Hubei province, in China, in December 2019, and has since evolved into a pandemic that has quickly spread throughout the world.^1^ It has caused social, economic and, especially, public health impacts in different countries. There are, to date, no drugs with proven efficacy for immunization against or treatment of this disease. Thus, extreme isolation measures, such as social distancing and “quarantine” have been the main strategies implemented worldwide to contain its propagation and to avoid collapse of health systems.^2 , 3^

Despite the benefits of social isolation on an epidemiological level, these measures have been associated with several negative psychosocial outcomes, such as depressive symptoms, higher levels of alcoholic beverage consumption, anxiety, post-traumatic stress disorder, feelings of loneliness, and problematic use of technology.^4^ Use of psychoactive substances as well as other rewarding behaviors (like gambling, playing videogames, and watching pornography) are frequently resorted to in attempts to reduce stress and anxiety and/or alleviate depressive moods.^5^ It is likely that there is a tendency towards substance abuse and towards engaging in these behaviors in an excessive manner as putative coping strategies in crises such as the COVID-19 pandemic.^5^

Faced with social distancing, information and communication technologies (ICT) have been employed in solutions to mitigate the impact of the pandemic, enabling implementation of health care initiatives involving infection control, diagnosis, and triage.^6 , 7^ ICTs are also a safe option for resuming social contact and academic and professional activities as well as for providing leisure and entertainment that may help lessen side effects of social distancing.^8^ As a result, there has been an increase in usage of ICTs during the COVID-19 pandemic. Anecdotal reports and little other scientific evidence indicate that an increase in online pornography use is expected likewise.^9 - 11^

With the rise in online pornography consumption, it is likely that there is also an increase in compulsive sexual behaviors and excessive masturbation.^10 , 11^ Data from Pornhub website show an increase of 11.6% in views in comparison to a random day before COVID-19 pandemic. In Brazil, there has been an even greater increase in views (13.1%).

Although, for most people, watching online pornography is adaptive and should not be pathologized, a subgroup of vulnerable individuals is at risk of developing problematic patterns of consumption.^12^ Frequent consumption of pornography, especially online, may lead to a range of side effects, such as lower levels of well-being, reduced productivity, and impaired interpersonal relationships, together with increased isolation and narrowing of social repertory, which add to the effects of the pandemic and the “quarantine.”^13^

In spite of the scarcity of more robust scientific data and the controversy around the subject (sometimes understood as harmless entertainment or benign sexual expression, other times seen as having harmful effects on society), recent studies estimate that pornography may gain new contexts and challenges in some parts of the world during and after the pandemic.^10 , 13 , 14^ In this sense, the aim of this study is to appraise problematic consumption of online pornography during the COVID-19 pandemic and suggest clinical recommendations on the matter.

## Clinical features

Several behaviors which potentially affect reward circuits in the human brain may lead to loss of control and may contribute to development of other symptoms of dependence in some individuals.^2 , 15^ Regarding internet dependence, neuroscientific research supports the assumption that the underlying neural processes are similar to those of addiction to psychoactive substances.^16^ The American Psychiatric Association (APA) acknowledges, in its 2013 revision of the Diagnostic and Statistical Manual of Mental Health Disorders, that one of these internet-related behaviors, online gaming, is a potential disorder which leads to dependence and merits further study.^17^

On the other hand, other internet-related behaviors, such as online pornography consumption, were not included. Nonetheless, there is little evidence that considers these behaviors as causes of dependence or addiction.^15 , 18^ In this context, “pornography dependence” is not a formally recognized disorder, mainly because of disagreements among researchers regarding the concept and clinical features and even its existence.^12 , 15 , 19 , 20^

However, self-perceived pornography dependence and its perception by health professionals and some researchers, which emerged in the 1970s, has become more relevant since the 1990s as a concept in research as well as in clinical practice. There is an alert on problematic consumption of pornography - its frequency and its possible side effects in legal, psychological (psychological and interrelationship suffering), and health-related aspects of people’s lives - since these individuals have been seeking help for dealing with such behaviors.^10 , 12^

In the United States of America (USA), previous work using several sampling techniques, like online convenience and grading samples, consistently shows that some pornography users report feeling “out of control” or “dysregulated” in their use of pornographic material. Nevertheless, there is still a lack of research with representative samples and adequate methods in different countries to examine the prevalence of this phenomenon of addiction to pornography.^20^ A study that evaluated 2075 adult internet users found that most participants had watched pornography at some point in life (n = 1,461), while a little more than half of them reported having done so in the year preceding the research (n = 1,056). Furthermore, approximately 11% of men and 3% of women reported some agreement with the statement “I am addicted to pornography.”^20^

Phenomenologically, behaviorally addicted individuals, such as pornography consumers, frequently show a problematic model of consumption, characterized by: a) impaired control (for example, unsuccessful attempts to reduce this behavior), b) compromise (such as narrow range of interests or neglecting other areas in life due to pornography consumption) and c) risky usage (persistent consumption, despite being conscious of harmful psychological effects). It remains arguable whether these behaviors also meet physiological criteria related to addiction (tolerance, abstinence).^10 , 12^

Individuals who frequently access pornographic content present compulsive sexual behavior (CSB), which is characterized by a pattern of sexual compulsion that frequently includes excessive masturbation, besides exaggerated sexual activity.^21^ Regarding comorbidities, the following can be found in association in individuals with CSB: anxiety and mood disorders, substance abuse, and erectile dysfunction.^12^

From a psychological point of view, affected individuals tend to show intense apprehension, feelings of guilt, and anguish.^21^ Most of these feelings can be influenced by religious and spiritual beliefs and also by moral concepts from the different societies in which these people live. A person’ perception of internet pornography dependence seems to be a predictor of interpersonal disagreements and religious and spiritual arguments.^19 , 20^ It has been highlighted that attitudes, beliefs, and behaviors shaped by pornography consumption have a deep impact on users’ private lives, as well as on professional and social relationships.^19 , 20^

Excessive consumption of pornography may impact users’ financial lives, involving them in legal and occupational problems and leading to interpersonal relationship difficulties.^10 , 12^ Another impact that can affect pornography users is sexual frustration related to their real-life sex partner.^22^ These users frequently criticize their sex partner’s body and pressure them to engage in high performance sexual acts, far removed from “real sex” and mostly fanciful.^22 , 23^

Although some available studies show little association between pornography and erectile dysfunction among young men,^24^ other researchers state that the rise in pornography consumption may be the main factor to explain the sharp increase in erectile dysfunction among young people.^25^ Another study points out that 60% of men who experienced erectile dysfunction with a real partner did not have the same problem when consuming internet pornography, raising the hypothesis of a complex background linked to the available and easily accessible world of internet sex.^26^

Individuals who consume pornography tend to be more susceptible to stress, impairing their ability to meet domestic demands, for example.^27 , 28^ If these subjects are regular users of online pornography and also parents, the impact may be even greater. Social isolation per se generates relevant negative outcomes in mental health,^4^ while young people tend to isolate even further into themselves during quarantine and surf the internet by themselves or with virtual friends, which may become an important source of stress due to the large number of hours that they remain connected.^29^

## Clinical recommendations

### In the context of children

One very pertinent concern that has been posited by researchers and also by the authors of this document refers to children and teenagers. Our opinion is that they deserve special attention, mainly because this is the period in which an individual’s identity develops and consolidates, as well as because it is the transition to adult life. Next, some very illuminating studies will be mentioned, since they may provide some insights into these concerns, especially with regards to young men.

The authors of this study highlight that over the course of the pandemic, children and teenagers seem to be more vulnerable in terms of the availability of Hardcore pornographic content, which is spread around the internet, and some studies seem to corroborate this hypothesis.^30 - 32^ First exposure to pornographic content at a premature age is a cause of concern for several different sectors of society nowadays. The importance and relevance of these data seem to be confirmed by several scientific studies. A study conducted with young people aged 15 to 29 years reported that mean age of first exposure to online pornography was at 13 for men and 16 for women.^33^ Moreover, 84% of those who had watched online pornography in the 12 months prior to the survey were men and 19% were women, but one curious fact was that women consumed pornography on a weekly or daily basis.^34^ Another study showed that 64% of young people aged from 13 to 24 actively sought pornography on a weekly basis.^33^ Research shows that children and teenagers are more inclined to problematic consumption of online pornography than adults.^35^

The most popular pornographic content website - Pornhub - reports that people watched 4.6 billion hours of pornography on the website’s digital platform in 2016,^36^ with 61% of visits being via smartphones.^36^ These websites’ popularity among the young public even outweighs other known sources of entertainment such as eBay, MSN, and Netflix.^36^

A peer-reviewed study of literature published in English-language journals, between 1995 and 2015, suggested that teenagers who watch online pornography may live with other problematic members of the family, with whom emotional bonds are weak or almost inexistent. Consumption of pornography was more prevalent in the male sex and was associated with stereotyped sexual attitudes and risky behavior like condomless sex and also appeared to be associated with sexually aggressive behaviors by those who practice penetration without consent, but also by others who suffer sexual and psychological violence in the context of victimization.^37^

A multi-centered study in Europe involving 4,564 teenagers between 14 and 17 years of age suggested that watching pornography on a weekly basis is associated with a higher probability of sending sexual content (sexting) between peers.^38^ In another systematic review and meta-analysis of 14 papers reporting cross-sectional studies, sexting behavior among people between 10 and 24 years of age was strongly associated with premature sexual activities, with alcohol use before the sexual act, and with multiple sex partners.^39^

Adolescence is a period in which experimentation with sexuality starts. According to specialists, sex education should begin in puberty and adolescence.^40^ Studies have already shown the inefficacy of sex education based on fear and guilt.^41^ Nonetheless, recently a new way of experimenting with sexuality through sexting, especially among pre-adolescent and adolescent populations, has come to the attention of parents, educators, psychologists, and the general public.^42^ Based on studies which suggest that online pornography consumption by young people is related to sexting, it is very likely that this practice will grow exponentially during the pandemic, since many young people and adults will spend more time online. Thus, the recommendation is to establish a dialogue between adults, adolescent, and preadolescent children about good and effective sex education, particularly during the period in which the world is dealing with COVID-19, and also warn about the possible outcomes from such behavior. Even though the aim of this document is in no way to recommend sexual activities among preadolescent and adolescent people, the authors of the study acknowledge that young people at this stage of development are curious about sexuality and it is necessary to differentiate between what is part of their development and what are the consequences of online pornography consumption and sexting.

We list some recommendations on the subject based on scientific evidence:

If any young person receives sexual content (sexting) during quarantine, it is advisable not to show it to anyone else, especially if there is a minor in the image - this may entail non-consensual sharing.^43^

There are fake profiles on apps such as Tik Tok, for example, through which people known as sexual predators encourage young people to engage in sexting.^44^

When sending any picture, if it is not known whether the person who will receive it is trustworthy, it is advisable not to send pictures containing intimate parts of the body or the person’s own face. Some social media websites contain sophisticated facial recognition algorithms that identify you automatically in all of the photos you wish to be kept secret.^45^

Verify with young people whether images which are shared on social media show tattoos, birthmarks, or other resources which could connect the picture to the sender. Likewise, it is ideal to disable the location mechanism of digital gadgets such as tablets, smartphones and laptops, in order not to be tracked by malicious people.^45^

If a young person is being coerced into sending naked pictures, advise him or her to collect as much evidence as possible, such as texts and pictures, and give them to their legal guardian to present them to protection services.

If a young person has sent a personal image, suggest using eraser applications immediately after sending the messages and visual content.^45^

Be careful to immediately delete any explicit photos or videos from the device. This applies to images sent by or received by the person. Having images stored on one’s own devices increases the probability of someone such as a hacker finding them. Possession of naked images of minors may have criminal implications. In 2015, for example, an adolescent from North Carolina, in the USA, was accused of possessing child pornography, even though the image on his phone was of himself.^45 , 46^

### In the context of adults

There is still no consensus in the literature on whether consumption of pornography is problematic or not and in particular there are no studies covering the current pandemic situation and online pornography consumption by adults and teenagers. A more recent study suggests that problematic online pornography use may be behind compulsive sexual behavior (CSB).^47^

Dhuffar & Griffiths found higher scores for sexual dependence and openness to sexual experiences in men than in women, among 267 participants. These authors preliminarily concluded that gender differences and related personality traits significantly contributed to this sexual dependence variance. They also pointed out that when women need to seek specialized help for this issue, the barriers are evident and many times hard to overcome – the main one is the sexist culture still present in our modern society, preventing women from been understood instead of criticized.^48^

Currently, there is scientific evidence revealing the intense impact of online pornography on adults’ sex lives and physical and mental health. Social media have largely contributed to the extent of this new form of entertainment, as the pornography industry likes to call itself. Young adults seem to have easy access, as the advent of high speed internet brought online pornographic content to users, mainly through social media and easy access websites.^49^

Regarding the internet, especially with the new information and communication technologies mentioned in this document, it is reported that this resource brings about behaviors seen as problematic, mostly on-line pornography consumption. The ease of finding pornographic content through social media and websites with high resolution movies for free may stimulate potential users to risky behavior. Problematic users who consume on-line pornography, may engage in multiple sexual gratification behaviors in the virtual world such as: online sex or cybersex, watching online porn, joining sexual chats, having sex on webcams with masturbation and fetishes, as well as tirelessly seeking new sexual partnerships, besides accessing pornographic movies watched by users in the traditional solitary manner.^49^

In the United States, for example, a study with a representative sample of adults suggests that approximately 46% of male public and 16% of females, reported having accessed online pornographic content in a programmed way and with the following outcomes, according to the study: possible addiction to pornographic content, mental suffering, and difficulties in connecting to religious or spiritual activities.^50^ The authors report that some psychological and social phenomena present in our modern society are related to online pornography consumers. Researchers identified, for instance, higher levels of loneliness, difficulties with inhibitory control, lower frequency of religious activities, and subjective feelings of greater dependence on online pornography.^50^

### Diagnostic criteria

Some scientific publications demonstrate that assiduous consumers of online porn are highly likely to develop sexual dependence over the years. Nevertheless, it is still a great challenge for scientific investigators and even clinicians of great expertise to deal with this issue categorical and multidimensionally. The American Psychiatric Association in its fifth edition of the Diagnostic and Statistical Manual of Mental Disorders (DSM-5) uses the term hypersexuality for one of the symptoms. However, although the DSM-5 has coined this term, it is difficult to identify a precise definition for the well-defined diagnostic and nomenclature criteria within the specialized literature on the theme. For example, sex dependence is imprecise, because this name comes from sex drive or desire, and not from bizarre sexual desires. Moreover, sex dependence may have different presentations which are not included in this term.^48^

There are other researchers who seem to better clarify the complexity of these concepts. According to De Alarcón et al., the term hypersexual sometimes includes repetitive habits and is employed in a broad definition that encompasses a range of problematic behaviors, such as cybersex, pornography use, excessively frequent masturbation, non-consensual sexual behaviors among adults, sex via the phone, visits to strip-tease clubs, and others.^10^ Prevalence rates vary from 3 to 6% - they are hard to predict since there is no structured definition of the disorder. Authors also point out that the scarcity of scientific data hampers research on the subject, as well as its conceptualization and evaluation, accepting a range of proposals to try to explain it. Hypersexual behavior is usually associated with significant suffering, with feelings of embarrassment and with self-censoring. Constant and intense engagement in sexual behaviors may strengthen the so called “response appetite”, simultaneously increasing the frequency of automatic responses and weakening impulse control. In summary, behaviors related to sexual impulses may be easily unleashed, requiring little effort to be initiated and, thus, may become hard to control, and regulated mainly on an unconscious level.^51^

### Evaluation and treatment

When compulsive sexual behavior and online pornography consumption in adults has lasted some time, even if the individual presents significant impairments, specialists recommend a solid evaluation of the case so that medical and specialized psychological aid can be offered and the most effective treatment can be planned.^48^

Regarding psychometric evaluation, the specialized literature presents a number of scales for assessing components which clinicians and researchers have identified as important, mainly when there is suspicion of problematic use of online pornography. There follows a summary of some scales and inventories and their respective descriptions and uses for evaluation of problematic consumption of online pornography. Compulsive pornography behavior, with the Compulsive Pornography Consumption (CPC) scale and the Cyberporn Compulsivity Scale (CCS); hypersexual behavior with the Pornography Consumption Inventory (PCI), developed to assess motivations for porn consumption exclusively among men with hypersexual behavior; deficient self-regulation with the Habit Strength Scale, Deficient Self-Regulation Scale, and Negative Consequences Scale – these come from a hypothetic model which investigated different elements of internet use and tried to conceptualize pornography dependence based on social-cognitive theory, where dysregulated internet use is the main differentiator between addiction patterns.^52^

Some time ago, clinicians and researchers also developed the Brief Pornography Screener (BPS), which is a screening tool for measuring problematic use of pornography and loss of self-control. This is a 5-item self-report questionnaire developed to detect problematic pornography use among clinical and non-clinical samples. It may be useful for identifying individuals at risk of using pornography in a problematic way or who are about to use it as a vicarious form of gratification.^52^

It is important to highlight that based on scientific literature, when considering the characteristics of sexual behavior, the clinician or researcher observes the patterns of failure in impulse control or intense sexual impulses, the results of repetitive sexual behaviors, and impulse control disorders. The BPS considers the main component of problematic use of pornography to be the compulsion to use pornographic material.^49^

Specialized psychological treatment together with pharmacological treatment may help the patient both in reducing sexual impulses and in developing coping strategies, creating a safe and trustworthy space to deal with the problem.^53^

Differential diagnosis evaluation may be of great importance as well, including Paraphilic Disorders, Bipolar Disorder, Internet Dependence, and Stimulant Drugs Dependence, which in general can make the individual more impulsive and hypersexualized, for example. It is imperative to include psychiatric comorbidities in the evaluation, especially Mood Disorders.^53^

The specialized literature does not indicate specific medication, but there are some psychotropic drugs capable of helping in the treatment process - Naltrexone targets reduction of compulsive sexual behavior. Selective serotonin reuptake inhibitors (SSRI) are a class of drugs employed in the treatment of depressive syndromes, anxiety disorders, and some kinds of personality disorders, and in high doses may contribute to treatment, especially for impulse control.^53^

Regarding group therapy, DASA (Dependentes de Amor e Sexo Anônimos) is a fraternity based on the 12-steps recovery program from the AA (Alcoholics Anonymous). DASA is a mutual help fraternity, open to everyone over 18 and of any sexual orientation. Its members include people who experience a compulsive need for sex and those with a desperate attachment to a specific person, and it has proved to be a good option for inclusion in psychosocial treatment packages, along with psychoactive drugs. Currently, DASA is available in online format.^53^

Specialized psychological support provided by psychologists or psychotherapist psychiatrists is of great importance. Motivational and cognitive-behavioral approaches have yielded evidence of good responses.^53^ Psychotherapy may help patients to deal with the feelings of shame, inadequacy, rage, and guilt related to problematic consumption of online pornography and possible repercussions such as losses in interpersonal relations and low productivity in work activities, for instance. The range of psychosocial intervention possibilities associated with psychotropic drugs will also aid these individuals in reducing impulses and help with low tolerance to stress, for example, helping them to involve themselves less in risky situations like sexual activities involving multiple partners.^53^

The current scenario of scientific studies on pornography is shaped by different theoretical perspectives. Without evidence to suggest one theoretical position as superior to the other, doctors and psychologists are at risk of recommending a treatment which is aligned to his or her theoretical view (or personal prejudices), but is not in accordance with the motivations which lead an individual to engage in specific sexual behaviors.^53^

## Conclusion

Online pornography consumption on the current scale is a risk for personal and social health, although one that may not yet be well acknowledged by health authorities. Thus, the authors encourage readers to be alert to and register these disorders and the consequent harm, especially during the COVID-19 epidemic.

Regarding children and teenagers, there are concerns that the consumption of online pornography and sexting may lead to long-term consequences such as: humiliation, extortion, victimization, segregation in the academic environment, important emotional problems, suicidal ideation, and even reputational damage.

The available scientific evidence in regard to pornography is still insufficient to identify a single definition of problematic use or dependence on pornography, since researchers in the area espouse conflicting ideas. There is a growing appeal in the scientific media for studies to be conducted that focus on the attempt to understand the contribution of problematic use of pornography in different contexts of digital societies. New studies are needed to assess the prevalence of pornography use, its patterns of consumption, as well as factors involved in persistent seeking of pornographic material, evaluating the possible link between this habit and failures of impulse control.

Specialist psychological treatment combined with drugs may help patients both to reduce sexual drive and to develop coping strategies, while offering a safe, trustworthy space to address the issue.

Scientific studies should be designed to better evaluate its diagnosis and repercussions, as well as to outline future preventive and therapeutic strategies for this population. Despite the age, we consider all the individuals mentioned in this manuscript merit the attention of the scientific community and civil society, in order to provide advice about the real benefits and risks of these practices.
